# Supplementary material for: An Inulin-Type Fructan CP-A from Codonopsis pilosula Alleviated 5-Fluorouracil-Induced Intestinal Mucositis via the ERK/MLCK/MLC2 Pathway and Regulation of Gut Microbiota
Source: Pharmaceuticals (Basel). 2024 Feb 26;17(3):297. doi: 10.3390/ph17030297 (PMC10975959; doi:10.3390/ph17030297)
Supplement: Supplementary file 1 [file pharmaceuticals-17-00297-s001.zip › pharmaceuticals-2809185-supplementary.pdf]

An Inulin-Type Fructan CP-A from *Codonopsis pilosula* Alleviated 5-Fluorouracil-Induced Intestinal Mucositis via ERK/MLCK/MLC2 Pathway and Regulation of Gut Microbiota

Jiangtao Zhou [1,2,3,†], Deyun Li [1,†], Jiajing Wang [1], Zhuoyang Cheng [1,2,3], Changjian Wang [1], Xuepeng Zhang [1], Xiexin Xu [1], Jianping Gao [1,2,3,*]

1 School of Pharmacy, Shanxi Medical University, Taiyuan 030001, China

2 Medicinal Basic Research Innovation Center of Chronic Kidney Disease, Ministry of Education, Shanxi Medical University, Taiyuan 030001, China

3 Shanxi Provincial Key Laboratory of Drug Synthesis and Novel Pharmaceutical Preparation Technology, Shanxi Medical University, Taiyuan 030001, China

* Corresponding: jpgao123@163.com

† These authors contributed equally to this work

Table S1 Sequences of the primers used in the RT-PCR

Gene	Primer	Gene sequence (5'-3')
IL-4	Forward	ACA AGG AAC ACC ACG GAG AAC G
	Reverse	TCT TCA AGC ACG GAG GTA CAT CAC
IL-6	Forward	GCC TTC TTG GGA CTG ATG TTG TTG
	Reverse	GTC TGT TGT GGG TGG TAT CCT CTG
IL-10	Forward	AAG GCA GTG GAG CAG GTG AAG
	Reverse	TGA GTA TCA CGT AGG CTT CTA TGC
TNF-α	Forward	CCG AGA TGT GGA ACT GGC AGA G
	Reverse	CCA CGA GCA GGA ATG AGA AGA GG
ERK1/2	Forward	GTT CCC AAA CGC TGA CTC CAA AG
	Reverse	GCC AGA GCC TGT TCA ACT TCA ATC
MLCK	Forward	TTC AAG ATG GTG GTG GCT GTG G
	Reverse	TGC TTG CTC CTT GTT CTC CTC AG

Table S2 Relative abundance of the most representative phyla in experimental rats

Phylum	Control	5-FU	BTC	CP-A L	CP-A M	CP-A H	Variation (5-FU)
	Relative abundance (%)						
Firmicutes	89.49±2.13**	62.86±12.86	80.66±3.20*	85.25±4.69*	86.32±2.08**	89.90±2.01**	↓
Proteobacteria	2.00±0.14**	27.03±11.78	7.58±3.80*	6.71±3.02*	4.50±1.14**	1.67±0.21**	↑
Bacteroidetes	6.97±1.74	6.54±2.54	9.95±2.23	3.24±1.29	5.26±2.09	6.63±1.83	↑
Actinobacteria	0.67±0.17	2.57±0.94	1.01±0.65	3.93±1.34	2.64±0.19	1.15±0.60	↑
Tenericutes	0.16±0.10	0.22±0.19	0.06±0.01	0.29±0.11	0.41±0.23	0.12±0.07	↑
Cyanobacteria	0.17±0.02	0.16±0.02	0.15±0.01	0.16±0.02	0.18±0.03	0.11±0.01	↓
Verrucomicrobia	0.02±0.01	0.02±0.01	0.02±0.01	0.03±0.02	0.06±0.02	0.01±0.01	↓
TM7	0.01±0.01	0.00±0.00	0.00±0.00	0.07±0.06	0.02±0.01	0.01±0.00	↓
Elusimicrobia	0.01±0.01	0.00±0.00	0.00±0.00	0.07±0.06*	0.02±0.01	0.01±0.00	↓
Firmicutes/ Bacteroidetes (F/B)	22.77±10.17	45.39±27.02	11.56±3.39	85.31±50.40	56.40±35.74	19.87±4.97	↑

Values were represented the mean±SEM.

Kruskal-Wallis test was applied for statistics and $^{*}p < 0.05$ and $^{**}p < 0.01$ versus 5-FU group (n = 6).

Table S3 Relative abundance of the most representative classes in experimental rats

Phylum	Class	Control	5-FU	BTC	CP-A L	CP-A M	CP-A H	Variation (5-FU)
		Relative abundance (%)						
Firmicutes	Clostridia	35.97±3.48	43.28±6.54	27.71±8.12	44.58±9.58	46.50±9.99	24.43±2.61	↑
	Bacilli	50.23±5.06**	13.38±5.85	50.28±10.24**	37.41±12.35*	30.32±7.72	61.39±4.20**	↓
	Erysipelotrichi	2.86±1.12	5.97±1.91	2.39±0.85	3.01±1.28	9.23±2.67	3.81±2.52	↑
Actinobacteria	Coriobacteriia	0.31±0.04*	1.99±0.89	0.89±0.66	1.18±0.53	0.85±0.19	0.57±0.29	↑
	Actinobacteria	0.35±0.15	0.57±0.49	0.12±0.03	2.75±1.45*	1.79±0.22	0.57±0.33	↑
Proteobacteria	Alphaproteobacteria	0.43±0.08**	0.22±0.02	0.24±0.02	0.28±0.03	0.32±0.04	0.21±0.01	↓
	Betaproteobacteria	0.42±0.04	0.25±0.03	0.28±0.03	0.52±0.21	0.37±0.06	0.18±0.02	↓
	Gammaproteobacteria	0.59±0.08**	25.44±11.94	6.03±3.42*	1.95±0.63**	2.47±1.37**	0.74±0.14**	↑
	Deltaproteobacteria	0.53±0.14	0.99±0.28	0.95±0.33	3.92±2.44	1.06±0.33	0.47±0.14	↑
Bacteroidetes	Bacteroidia	6.96±1.74	6.53±2.54	9.95±2.23	3.23±1.29	5.25±2.09	6.60±1.83	↓

Values were represented the mean±SEM.

Kruskal-Wallis test was applied for statistics and $^{*}p < 0.05$ and $^{**}p < 0.01$ versus 5-FU group (n = 6).

Table S4 Relative abundance of the most representative orders in experimental rats

Phylum	Order	Control	5-FU	BTC	CP-A L	CP-A M	CP-A H	Variation (5-FU)
		Relative abundance (%)						
Firmicutes	Clostridiales	35.97±3.48	43.27±6.54	27.70±8.12	44.58±9.58	46.49±9.99	24.43±2.61	↑
	Lactobacillales	50.07±5.08**	12.96±5.68	50.22±10.23**	37.23±12.36*	29.97±7.64	60.60±4.82**	↓
	Erysipelotrichales	2.86±1.12	5.97±1.91	2.39±0.85	3.01±1.28	9.23±2.67	3.81±2.52	↑
Proteobacteria	Desulfovibrionales	0.52±0.14	0.99±0.28	0.95±0.33	3.92±2.44	1.05±0.33	0.47±0.14	↑
	Burkholderiales	0.42±0.04	0.25±0.03	0.28±0.03	0.51±0.21	0.37±0.06	0.18±0.02	↓
	Enterobacteriales	0.27±0.08**	25.11±11.93	5.79±3.43*	1.33±0.69**	2.20±1.38**	0.45±0.16**	↑
	Pseudomonadales	0.28±0.02	0.23±0.03	0.20±0.03	0.57±0.33	0.21±0.04	0.22±0.04	↓
Actinobacteria	Coriobacteriales	0.31±0.04	1.99±0.89	0.89±0.66	1.18±0.53	0.85±0.19	0.57±0.29	↑
	Actinomycetales	0.32±0.16	0.50±0.45	0.07±0.01	2.71±1.45*	1.09±0.36	0.38±0.30	↑
Bacteroidetes	Bacteroidales	6.96±1.74	6.53±2.54	9.95±2.23	3.23±1.29	5.25±2.09	6.60±1.83	↓

Values were represented the mean±SEM.

Kruskal-Wallis test was applied for statistics and *P<0.05 and $^{**}p < 0.01$ versus 5-FU group (n = 6).

Table S5 Relative abundance of the most representative families in experimental rats

Phylum	Family	Control	5-FU	BTC	CP-A L	CP-A M	CP-A H	Variation (5-FU)
		Relative abundance (%)						
Firmicutes	Lactobacillaceae	49.29±5.19**	11.60±5.33	49.90±10.17**	33.05±13.36*	27.86±7.54	59.44±5.59**	↓
	S24-7	4.82±0.88	3.43±1.25	7.23±1.78*	1.97±0.53	3.77±1.26	5.45±1.54	↓
	Peptostreptococcaceae	2.84±0.97	4.52±2.36	2.32±0.97	5.73±1.55	3.70±1.09	2.39±0.63	↑
	Clostridiaceae	1.04±0.28	1.29±0.56	0.36±0.06	0.74±0.20	0.64±0.14	0.53±0.13	↑
	Erysipelotrichaceae	2.86±1.12	5.97±1.91	2.39±0.85	3.01±1.28	9.23±2.67	3.81±2.52	↑
	Ruminococcaceae	16.33±2.24	8.27±3.28	7.56±2.20	16.81±4.27	16.60±7.39	7.40±1.77	↓
	Lachnospiraceae	8.78±2.44*	23.20±6.25	11.74±5.17	11.55±4.13	17.21±5.44	9.08±1.16*	↑
Proteobacteria	Enterobacteriaceae	0.27±0.08**	25.11±11.93	5.79±3.43*	1.33±0.69**	2.20±1.38**	0.45±0.16**	↑
	Desulfovibrionaceae	0.52±0.14	0.99±0.28	0.94±0.33	3.92±2.44	1.05±0.33	0.47±0.14	↑
Actinobacteria	Coriobacteriaceae	0.31±0.04**	1.99±0.89	0.89±0.66	1.18±0.53	0.85±0.19	0.57±0.29	↑

Values were represented the mean±SEM.

Kruskal-Wallis test was applied for statistics and $^{*}p < 0.05$ and $^{**}p < 0.01$ versus 5-FU group (n = 6).

Table S6 Relative abundance of the most representative families in experimental rats

Phylum	Genus	Control	5-FU	BTC	CP-A L	CP-A M	CP-A H	Variation (5-FU)
		Relative abundance (%)						
Firmicutes	Lactobacillus	48.89±5.22**	11.34±5.21	49.42±10.08**	32.92±13.33	27.66±7.49	59.21±5.60**	↓
	Oscillospira	0.60±0.22	2.45±1.71	0.77±0.40	3.21±1.37	1.17±0.25	0.84±0.47	↑
	Blautia	4.19±1.69	10.69±5.44	3.06±0.89*	1.84±0.58*	4.68±1.70	3.72±0.97	↑
	Dorea	0.42±0.20	2.35±0.79	2.25±1.77	1.53±1.05	0.54±0.26	0.29±0.08	↑
	Ruminococcaceae_Ruminococcus	1.79±0.73*	0.39±0.16	1.22±0.48	0.69±0.25	0.77±0.18	0.87±0.26	↓
	Subdoligranulum	2.30±1.93	0.44±0.23	1.08±1.00	1.71±1.01	1.54±1.18	0.62±0.32	↓
Bacteroidetes	Bacteroides	0.10±0.03*	1.88±1.23	0.78±0.27	0.47±0.36	0.86±0.62	0.25±0.03	↑
Proteobacteria	Shigella	0.16±0.04**	24.25±11.64	5.45±3.33*	1.13±0.67**	2.00±1.34**	0.36±0.16**	↑
	Desulfovibrio	0.45±0.13	0.79±0.22	0.67±0.19	2.33±1.17*	0.91±0.29	0.38±0.12	↑
Tenericutes	Allobaculum	2.21±1.14	5.60±1.82	1.59±0.86	2.36±1.36	8.54±2.80	2.74±2.64	↑

Values were represented the mean±SEM.

Kruskal-Wallis test was applied for statistics and $^{*}p < 0.05$ and $^{**}p < 0.01$ versus 5-FU group (n = 6).
